# Sarcoma and the 100,000 Genomes Project: our experience and changes to practice

**DOI:** 10.1002/cjp2.174

**Published:** 2020-06-23

**Authors:** Sophie C Prendergast, Anna‐Christina Strobl, William Cross, Nischalan Pillay, Sandra J Strauss, Hongtao Ye, Daniel Lindsay, Roberto Tirabosco, Jane Chalker, Shazia S Mahamdallie, Alona Sosinsky, RNOH Pathology Laboratory and Biobank Team, Genomics England Research Consortium, Adrienne M Flanagan, Fernanda Amary

**Affiliations:** ^1^ Research Department of Pathology University College London Cancer Institute London UK; ^2^ Department of Histopathology Royal National Orthopaedic Hospital NHS Trust Stanmore UK; ^3^ Department of Oncology University College London Hospital NHS Foundation Trust London UK; ^4^ SHIMDS Acquired Genomics Great Ormond Street Hospital for Children NHS Trust London UK; ^5^ Rare and Inherited Disease Laboratory Great Ormond Street Hospital for Children NHS Trust London UK; ^6^ Genomics England London UK

**Keywords:** cancer, genetics, genome, sarcoma

## Abstract

The largest whole genome sequencing (WGS) endeavour involving cancer and rare diseases was initiated in the UK in 2015 and ran for 5 years. Despite its rarity, sarcoma ranked third overall among the number of patients' samples sent for sequencing. Herein, we recount the lessons learned by a specialist sarcoma centre that recruited close to 1000 patients to the project, so that we and others may learn from our experience. WGS data was generated from 597 patients, but samples from the remaining approximately 400 patients were not sequenced. This was largely accounted for by unsuitability due to extensive necrosis, secondary to neoadjuvant radiotherapy or chemotherapy, or being placed in formalin. The number of informative genomes produced was reduced further by a PCR amplification step. We showed that this loss of genomic data could be mitigated by sequencing whole genomes from needle core biopsies. Storage of resection specimens at 4 °C for up to 96 h overcame the challenge of freezing tissue out of hours including weekends. Removing access to formalin increased compliance to these storage arrangements. With over 70 different sarcoma subtypes described, WGS was a useful tool for refining diagnoses and identifying novel alterations. Genomes from 350 of the cohort of 597 patients were analysed in this study. Overall, diagnoses were modified for 3% of patients following review of the WGS findings. Continued refinement of the variant‐calling bioinformatic pipelines is required as not all alterations were identified when validated against histology and standard of care diagnostic tests. Further research is necessary to evaluate the impact of germline mutations in patients with sarcoma, and sarcomas with evidence of hypermutation. Despite 50% of the WGS exhibiting domain 1 alterations, the number of patients with sarcoma who were eligible for clinical trials remains small, highlighting the need to revaluate clinical trial design.

## Introduction

The 100,000 Genomes Project (100KGP) was announced by the UK Government in 2012 and represented a step forward in making genomic medicine a reality for the National Health Service (NHS). Whole genome sequencing (WGS) of 100,000 genomes from NHS patients with either cancer or a rare hereditary disease was planned, and the Project aimed to fulfil five main aims: (1) to establish a genomic medicine service for the NHS, (2) to provide clinical diagnosis with new personalised treatment options, (3) to enable scientific discovery, (4) to foster development of the growing UK genomics industry, and (5) to facilitate patient engagement with genomic medicine [1].

Genomics England Ltd. (GEL), a government‐owned company, was founded the following year to deliver the Project. Biotechnology Company Illumina® (San Diego, CA, USA) was commissioned to sequence the DNA of patients and deliver bioinformatic pipelines for identification and cataloguing of variants. Thirteen Genomic Medicine Centres, each with their own Genomic Laboratory Hub, were established in England to deliver the end‐to‐end pathway, from recruitment of patients to return of results. The Project commenced in January 2014 and the recruitment of patients was closed on the 31 December 2018.

Establishing WGS as a clinical support service within the NHS was a fundamental aim of the 100KGP. This paper describes the lessons learnt from our participation, as one hospital, in the Project and how our experience will eventually enable the implementation of genomic medicine, as standard practice, within sarcoma management.

## Materials and methods

### Process review

All components of the pre‐ and post‐analytical processes of the 100KGP for patients with sarcoma were reviewed. Diagnoses were classified according to the WHO Classification of Tumours of Soft Tissue and Bone [2]. The pre‐analytical process included the consenting and registration of patients, sample collection and processing, and clinical data collection.

The post‐analytical process included review of the sequencing results, discussion of these at the Genomic Tumour Advisory Board (GTAB), and reporting the final results to the clinicians under whose care the patient was at the time of recruitment. Clinical and pathology data were available from hospital information systems and the Genomics Networked Information Exchange platform [3].

### Recruitment

Patients with sarcoma from the Royal National Orthopaedic Hospital (RNOH), a member of the North Thames Genomic Medical Centre, were consented to participate during hospital visits. A minority of patients were offered the opportunity to consent via post or email, in conjunction with a telephone conversation from a suitable qualified person. Participation required agreeing to most elements of the project, including the feedback of pertinent clinical findings, although feedback of additional findings, including carrier testing, was optional.

A suspected or confirmed diagnosis of malignancy, on a biopsied or resected tumour sample, could be frozen and stored as part of the clinical pathway prior to consent, provided this did not hinder delivery of a safe and timely diagnosis and/or treatment. If the sample proved to be a sarcoma and was considered suitable for WGS, patients or guardians were subsequently approached to participate in the project. Mandatory matching blood samples for germline sequencing were also obtained [4] and a standardised set of demographical and clinical data was collected for each patient. Specimens were annotated with Standard PREanalytical Codes (see supplementary material, Supplementary materials and methods) [5].

### Sample selection and processing

Frozen sections were inspected for percentage of tumour necrosis, tumour content, and cellularity. Only samples reaching the minimum criteria defined by GEL (>40% of tumour cells; <20% of necrosis) proceeded to DNA extraction using a standardised protocol [6]. These criteria were employed irrespective of whether the tumour had been subjected to neoadjuvant therapy or was treatment naïve. For some patients, multiple samples were submitted, to study tumour evolution and heterogeneity (Table 1).

**Table 1:** Ten most common diagnoses and numbers of whole genomes generated.

Diagnosis	Number of patients consented	Number of patients whose genomes were sequenced	Number of genomes generated from biopsies*	Number of genomes (*n*) generated from resections*	Mean number of samples sent per patient (range)
Chondrosarcoma	183	105 (57%)	3	106	2 (1–6)
Osteosarcoma	141	85 (60%)	18	80	3 (1–8)
Myxofibrosarcoma	112	82 (73%)	12	73	2 (1–6)
Lipomatous tumours	85	51 (60%)	12	41	3 (1–5)
Leiomyosarcoma	63	32 (51%)	8	30	2 (1–4)
Undifferentiated pleomorphic sarcoma	46	32 (70%)	6	26	2 (1–5)
Ewing sarcoma	40	16 (40%)	13	5	1 (1–2)
Malignant peripheral nerve sheath tumour	36	17 (47%)	4	24	2 (1–4)
Synovial sarcoma	34	25 (74%)	3	23	2 (1–5)
Chordoma	29	20 (69%)	2	19	2 (1–5)
All other	188	132 (70%)	16	121	2 (1–5)
Total	957	597 (62%)	97	548	2.09

* 51 patients had both a biopsy and a resection specimen submitted for sequencing.

Samples from 34 patients failed the internal quality control process at the NHS Genomic Laboratory Hub, Great Ormond Street Hospital for Children NHS Foundation Trust. On re‐cutting material at RNOH, samples from 24 patients were ‘rescued’ and sequenced.

We modified the GEL sample handling procedure to include cutting additional serial tissue sections into TRIzol® for RNA sequencing (see supplementary material, Supplementary materials and methods) in the future. We also extracted more DNA than required for WGS, so that epigenetic studies could be performed in the future on the same nucleic acid. DNA extraction from blood was undertaken using standardised protocols. Generally, the plasma was separated from whole blood and stored for circulating tumour DNA assays to be developed. DNA from tumour and blood was sent to our NHS Genomic Laboratory Hub. DNA quality was then assessed and submitted to the UK Biobank for plating and additional quality assessment. That was followed by transfer of DNA to the Illumina® sequencing facility at the Wellcome Genome Campus, Cambridgeshire.

### Data analysis

Following WGS to ×100 average sequencing depth for tumours and ×30 for germlines, Illumina®'s North Star pipeline (version 2.6.53.23) was used for primary analysis (alignment and variant calling). Quality assessment of sequencing data, normalisation, flagging of potential false positives, functional annotation, and prioritisation of somatic variants as well as germline pertinent findings was performed through the automated pipeline at Genomics England [7] centrally.

Tumour and normal genomes aligned to the human reference genome and processed variant files were submitted in batches to the Genomics England Inuvika Research Environment. This is a secure, browser‐based, virtual desktop and computer space to which only registered academic and industry members have access. To ensure that planned research using these data complements the aims of the project and that researchers work collaboratively rather than duplicating efforts, multidisciplinary Genome Clinical Interpretation Partnerships [1] were formed. The aims and objectives of the collaboration were clearly defined and approved by a research access committee.

Clinical implications of WGS data were discussed at the new GTAB, held every 2 weeks for the North Thames Genomic Medical Centre, with a minimum representation of one clinical scientist, a pathologist and an oncologist. Somatic and germline alterations relevant to the disease and/or clinical scenario were discussed and interpreted as to whether they were clinically actionable.

## Results

### Cohort and consent

During the 5 years of the 100KGP, the RNOH diagnosed and treated 1256 patients with sarcoma, 861 (69%) of whom were approached to participate. Ninety‐four patients declined, although 69 of these individuals consented to participating in our Biobank for Health and Disease (National Research Ethics Service Committee Yorkshire and The Humber – Leeds East; 15/YH/0311; HTA license 12055) which involved providing tissue and clinical data for research. No patients withdrew their consent.

With permission from Genomics England, an additional 190 patients whose tumour samples had been stored prior to the start of the Project were recruited retrospectively. By the end of the recruiting period, 957 patients, including 533 males and 424 females, had been consented at the RNOH to take part.

The age of patients recruited ranged from 1 to 88 years old (average 46 years) including 76 paediatric patients, defined as those under the age of 16 years. In total, 538 patients had a soft tissue tumour and 419 had a bone tumour; 18 had a diagnosis of metastatic carcinoma and two had a diagnosis of lymphoma.

In total, 395 patients diagnosed with sarcoma were not approached for consent. The most common reasons for this included surgery that took place out of hours and at weekends, samples that were placed in formalin, and samples collected in other healthcare services.

### Sample success and failure

Of the 957 recruited patients, 597 (62%) had WGS results generated from their tumours. Of these, approximately 13% had at least one sample that was subjected to a reduced‐bias PCR amplification step (Illumina® TrueSeq DNA NANO) due to inadequate DNA concentration. The percentage of cases that were amplified was tumour biased; of 84 conventional cartilaginous tumours sequenced, 30 (36%) had at least one sample that was PCR amplified. This process generated sequencing data that was not reliable.

The most common reasons why samples were not suitable for WGS were:

#### Neoadjuvant therapies

In total, 288 (30%) of the 957 patients consented for the Project received neoadjuvant chemotherapy or radiotherapy, including 140 (39%) of the 360 patients whose samples were not sequenced. These samples on frozen section often showed more than 20% necrosis and/or less than 40% tumour content, disqualifying them (see supplementary material, Supplementary materials and methods). Patients with Ewing sarcoma, almost invariably treated with neoadjuvant chemotherapy, were among the least likely to have their genomes sequenced (Table 1).

#### Sample type

The majority of samples that underwent sequencing came from resection specimens. Biopsy samples were frozen from 226 patients, of which 126 had DNA extracted. Ninety‐seven (77%) of these passed quality control and generated informative whole genomes (Table 1). The use of biopsy samples enabled 48 patients to benefit from WGS who otherwise could not have, as their subsequent resection specimens were necrotic or otherwise unsuitable. Another 20 (9%) of the 226 frozen biopsies were thawed because a diagnosis could not be safely reached on the formalin‐fixed sample processed initially. The remaining 80 biopsy samples remain in storage for future studies.

#### Disease‐specific challenges

Disease‐specific factors affected the number of samples suitable for sequencing. For example, 39% of chondrosarcomas, including low and high‐grade disease, were not sequenced. Low grade cartilaginous tumours are paucicellular and matrix‐rich, often yielding insufficient DNA. By contrast, high grade tumours, not restricted to chondrosarcoma, are often extensively necrotic.

### Reasons for fresh tissue not being frozen

#### Gross inspection of resection specimens

Failure to freeze tumour was partly accounted for by inadequate training and experience of biomedical scientists and pathologists. Some specimens were considered too small despite having a minimum dimension of 20 mm [6]. Secondly, extensive haemorrhage of specimens and/or necrosis on gross inspection resulted in samples not being frozen. However, on microscopic assessment, 20 of 157 (13%) of these samples showed significant amounts of viable tumour, which if frozen would have likely resulted in adequate DNA for WGS (Table 2).

**Table 2:** Reasons for failure to submit DNA from resection specimens for WGS from 360 patients.

	Explanation	Number of patients whose genomes were not sequenced (%)
Frozen tissue from resection specimens not available (195 patients)	Judged not suitable for freezing on gross inspection: small tumour size or extensive necrosis	157 (80%): (100 post neoadjuvant therapy)
	Resection specimen received in formalin	27 (14%)
	Other	11 (6%)
DNA from frozen resection specimens not sequenced (165 patients)	Failed microscopy assessment (GEL criteria): low cellularity, extensive necrosis	83 (50%): (47 post neoadjuvant therapy)
	Failed DNA extraction QC having passed microscopy assessment. Mainly accounted for by paucicellular tumours (Table 1)	45 (27%)
	No normal control for germline sequencing	9 (5%)
	Failed GEL internal QC [6]	7 (4%)
	Other	21 (13%)

#### Referral of patients post diagnosis

Many patients with sarcoma are diagnosed outside specialist services. Between January and December 2017, 289 patients were referred to our pathology tertiary referral service having already had surgery and having not been approached to participate in the project. The referrals came from 52 different NHS Trusts, ranging from tertiary referral centres to district general hospitals (Table 3).

**Table 3:** Patients referred to our unit post‐surgery for pathology review and clinical management: most common anatomical sites involved.

Anatomical location	Number of cases (*n* = 289)
Abdominal and pelvic visceral organs	53
Gynaecological tract	42
CNS, skull, spinal column	43
Head and neck	35
Thorax, thoracic visceral organs	26
Skin and scalp	27
Breast	17
Lower urinary tract	2
Unknown	4
Bone and soft tissue sarcoma (sites other than above)	40

#### Out‐of‐hours surgery

When surgery took place at the weekends and operations finished outside the opening times of the Pathology Department, samples were fixed in formalin.

### The Genomic Tumour Advisory Board

In this study, we reviewed the WGS results from the first 350 patients whose results have been analysed and discussed at our GTAB (see supplementary material, Table S1).

#### Correlation of WGS with current standard diagnostic testing

Approximately one third of sarcomas are defined by a recurrent genetic alteration [2]. Detection of these alterations using diagnostic tests such as immunohistochemistry, fluorescent *in situ* hybridisation (FISH), and PCR‐based tests was confirmed by WGS in the majority of cases (Table 4). Bioinformatic tools employed by Illumina®'s pipeline were more successful in confirming single nucleotide variants than structural alterations. Specifically, amplification of *MDM2*, which characterises both well‐differentiated and dedifferentiated liposarcoma [8] and parosteal osteosarcoma [9] and is routinely detected by FISH, were not called by the Canvas algorithm for copy number variant calling. The *TBXT* low copy number gain [10] was also not called using the WGS automated pipelines. These cases were reported to GEL and used for training of the next generation of the analytical pipeline. *SS18* rearrangements were only detected if the fusion partner was *SSX1* but not if *SSX2* [11] (Table 4). The failure to call breakpoints in *SSX2* is explained by their location in the region of segmental duplication and must be accepted as a limitation of short‐read WGS.

**Table 4:** Correlation of standard of care testing with WGS.

Histological main diagnosis	GTAB cases (*n*)	Expected hallmark genetic alteration	Alteration detected by standard of care testing (*n*)	WGS confirmed expected alteration (*n*)	Concordance between WGS and standard of care testing (%)
Alveolar soft part sarcoma	3	*ASPSCRA‐TFE3*	FISH (2)	2	100
Angiomatoid fibrous histiocytoma	1	*EWSR1‐CREB1* *EWSR1‐ATF1*	FISH (1)	1	100
Chondrosarcoma (conventional central)	47	*IDH1* (27) *IDH2* (7)	ddPCR (33), Sanger sequencing (1)	34	100
Extraskeletal chondrosarcoma	5	*EWSR1‐NR4A3* *TAF15‐NR4A3*	FISH (4)	5	80
Mesenchymal chondrosarcoma	7	*HEY1‐NCOA2*	RT‐PCR (4)	0*	0
Chondrosarcoma (peripheral)	3	*EXT1*, *EXT2* loss	NA	3	NA
Chordoma	15	*TBXT* gain	FISH (1), IHC (14)	4*	27
Clear cell sarcoma	5	*EWSR‐ATF1* *EWSR1‐CREB1*	FISH (5)	5	100
Dermatofibrosarcoma protuberans	3	*COL1A1‐PDGFB*	FISH (3)	3	100
Epithelioid sarcoma	5	*SMARCB1* loss	IHC (5)	4	80
Ewing sarcoma	14	*EWSR1‐*rearrangement	FISH (14)	13	93
Low grade fibromyxoid sarcoma	7	*FUS‐CREB3L2*	IHC (7)	7	100
Chondrosarcoma arising in fibrous dysplasia	2	*GNAS1*	ddPCR (2)	2	100
Giant cell tumour of bone	3	*H3F3A* (G34W)	IHC, ddPCR (both x3)	2	67
Leiomyosarcoma	24	*MYOCD* gain	NA	5	NA
Liposarcoma (dedifferentiated)	6	*MDM2* amplification	FISH (6)	0*	0
Liposarcoma (myxoid, round cell)	3	*FUS‐DDIT3*	FISH (3)	3	100
Malignant solitary fibrous tumour	4	*NAB2‐STAT6*	IHC (4)	4	100
MPNST	6	*NF1* pathogenic germline mutation	NA	2	NA
Osteosarcoma (parosteal)	7	*MDM2* amplification	FISH (7)	1*	14
Rhabdomyosarcoma (alveolar)	1	*FOXO1‐PAX3* *FOXO1‐PAX7*	FISH (1)	1	100
Synovial sarcoma	19	*SS18‐SSX2* *SS18‐SSX1*	FISH (19)	13*	68
Undifferentiated pleomorphic sarcoma	13	*MED12‐PRDM10*	NA	1	NA

ddPCR, droplet digital PCR.

* *SS18‐SSX2* fusion, *HEY1‐NCOA2* fusion and *TBXT gain* and *MDM2* amplification not detected through WGS.

#### New discoveries and refinement of diagnoses

**Figure 1:**
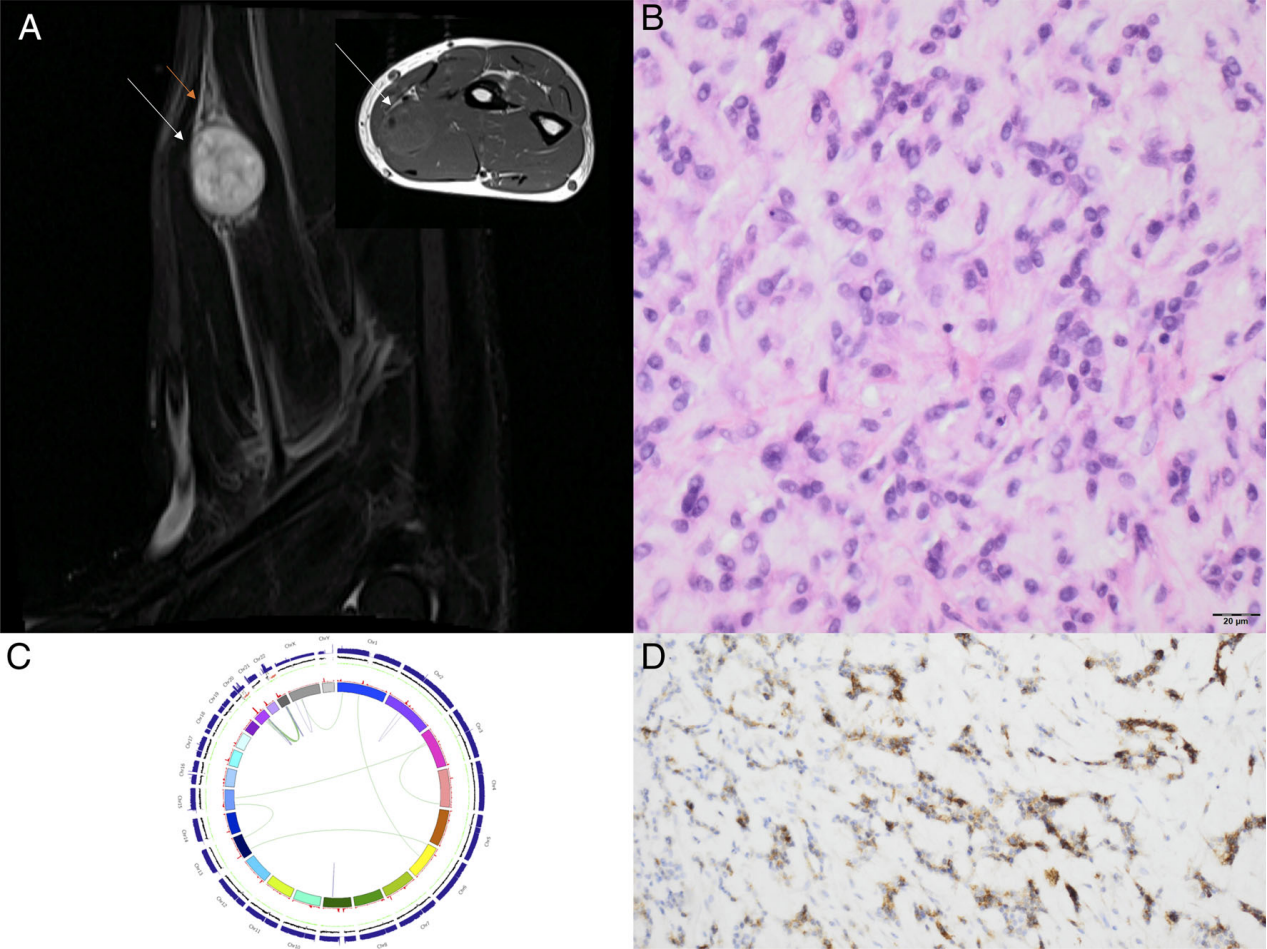
*EWSR1‐NFATC2* rearranged sarcoma arising from a nerve. (A) T1 coronal and axial (insert) MRI scan showing a tumour (white arrows) arising from the radial nerve (red arrow). (B) Photomicrograph of haematoxylin and eosin‐stained section showing a round‐cell tumour. (C) Circos plot showing the WGS‐detected rearrangement between *EWSR1* on chromosome 22q12.2 and *NFACT2* on chromosome 20q13.2 (D) Photomicrograph of section of tumour showing EMA expression.

Of the cohort of 350 patients discussed to date, we have identified findings in the WGS which required modification of the original histopathological diagnosis in 8 patients overall (3%) (see supplementary material, Table S1). One tumour diagnosed as a malignant peripheral nerve sheath tumour (MPNST) revealed an ultra‐violet light hypermutator signature [12, 13] and was reclassified as malignant melanoma. A second MPNST was reclassified as fibrosarcomatous transformation of a dermatofibrosarcoma protuberans on discovery of the characteristic *COL1A1‐PDGFB* fusion [14]. Two cases revealed an *EWSR1‐NFATC2* fusion [15] using the WGS calling pipeline; one had been diagnosed as an Ewing sarcoma and the other as an unusual sarcoma not otherwise specified arising from a nerve (Figure 1). Two chondrosarcomas reported as secondary to osteochondroma, peripheral subtype, were reclassified as central following detection of somatic *IDH1/2* variants [16]. The final case was a primary bone tumour harbouring an *EWSR1‐SMAD3* fusion, an abnormality not previously reported in bone [17].

#### Clinically relevant genetic alterations and clinical trials

Domain 1 variants are somatic single nucleotide variants and indels less than 50 base pairs that are not disease‐specific and which have reported therapeutic, prognostic or clinical trial associations, as defined by the GenomOncology Knowledge Management System [18]. Domain 2 variants are cancer‐related alterations as defined by the Cancer Gene Census [19]. An alteration in a domain 1 gene was detected in approximately 55% of cases, including cases of chondrosarcoma, myxofibrosarcoma, and osteosarcoma, which are among the most common sarcoma subtypes submitted for WGS (see supplementary material, Table S1). The most frequently altered domain 1 genes included *TP53*, which was detected in 52 tumours, *IDH1* or *IDH2*, detected in 35 tumours and *ATRX*, detected in 25 tumours (see supplementary material, Table S1). All domain 1 and 2 variants were discussed at our GTAB.

Tumour mutational burden can be used as a biomarker to stratify patients for treatment with checkpoint inhibitors [20]. A consensus on what defines a high mutational burden is currently lacking [21], but in sarcoma, this has been proposed to be >5 mutations/megabase. Within our patient cohort of 350, 8 (3%) tumours had this finding.

#### Germline findings

Germline variants are sought using cancer susceptibility gene panels, regularly updated by Genomics England [22]. The germline genomes of patients were assigned to the relevant gene panel based on their enrolled cancer type. When detected, germline variants are tiered based on their clinical relevance and pathogenicity [7]. Tier 1 variants are considered to be pathogenic or likely pathogenic variants for the patient's enrolled cancer type. Tier 3 includes rare variants reported in genes in a broader set of cancer susceptibility panels not currently associated with sarcoma, e.g. *BRCA1*. Tier 3 variants are reviewed by clinical scientists, but only discussed at GTAB and actioned if pertinent to the patient's disease type or their family's cancer history, such as *NF1* germline alterations detected in individuals with MPNST (see supplementary material, Table S1).

All pathogenic and likely pathogenic germline variants [23] detected in our 350 patients were discussed at our sarcoma multidisciplinary meetings (MDMs) which involved input from a clinical geneticist. In total, six patients (four adults and two children) had a tier 1 germline alteration. Each patient harboured a likely pathogenic *TP53* alteration, two of whom were paediatric patients with osteosarcoma [24]. All germline alterations detected in children are also studied using a childhood cancer panel. As a result, we discussed selected paediatric patients with variants classified as being of ‘uncertain pathogenic significance’ [23] including two children with germline alterations in *SQSTM1* and *TSC2* (see supplementary material, Table S1).

## Discussion

### Sarcoma: rare, but well represented

Sarcoma represents 1% of all cancers [25], with approximately 3700 new sarcomas diagnosed annually in the UK [26]. Despite this, the number of patients with sarcoma whose samples were sent for sequencing as part of the 100KGP ranked third highest overall. Only patients with breast and colorectal cancers (representing respectively 55 000 and 42 000 new cancers diagnosed annually in the UK [27]) had a higher number of sample submissions.

### Changing clinical pathways

As a research‐active pathology department, we adjusted readily to the demands of the 100KGP, a finding seen in other research‐active units across the country. Nonetheless, several changes were introduced to increase patient recruitment. Fresh resection samples could be stored in a fridge/cold room (4 °C) for up to 96 hours without significant DNA degradation or loss of quality of histological features [28]. Initially, some patients could not be recruited to the project as their samples were being fixed in formalin. Removal of formalin from the operating theatres also proved transformative in increasing patient eligibility for the Project.

Initially, we froze material from resected specimens in preference to biopsy samples for WGS as this was more time efficient. However, our finding that there was insufficient material of the quality stipulated by GEL in 40% of cases that had been subjected to neoadjuvant therapies underscores the need to submit biopsy material where feasible for WGS. Nonetheless, sequencing of viable post‐therapy material is also valuable as it reveals the genetic changes that are resistant to the therapy received.

### Trials and therapies

A major aim of the 100KGP was to identify patients with actionable genetic alterations for which they could receive existing therapies and/or be recruited to early phase clinical trials. Although over 50% of our patients had an alteration in domain 1, only a minority were found in a gene that is associated with an approved clinical therapy or that can be used as a prognostic biomarker [29]. The majority of these variants occurred in *TP53* and *ATRX*, which have potential clinical significance and are associated with open early phase clinical trials [30] but remain for the moment non‐actionable for patients with sarcoma. The majority of our patients were in remission directly post‐surgery and therefore the genetic findings were not clinically relevant. In the event of relapse, the genomic findings could still provide opportunities for patients to access novel therapies.

For patients with active disease, it was disappointing that only a single clinical trial was open to the recruitment of patients with sarcoma and that this was limited to surgically untreatable chondrosarcomas with somatic *IDH1* mutations [31]. As yet, sarcoma is infrequently among the cancer types included in clinical trial designs due to its rarity. Our findings underscore the need to design clinical trials in new ways that will ultimately make drugs more widely available to patients with sarcoma and other rare cancers. In particular, patients with sarcoma with a high mutational burden should be included in the relevant immune checkpoint blockage clinical trials going forward.

### Improved classification of disease

Sarcoma comprises approximately 70 different subtypes. Although a diagnostic molecular hallmark exists for many, providing an accurate diagnosis remains a challenge in some sarcoma subtypes, as highlighted by The Cancer Genome Atlas publication [32]. Among our cohort of 350 whose WGS results have been discussed, we altered the diagnosis for no more than 3% of patients, but this feedback is already making us more critical when reporting diagnoses. No modification of clinical management was indicated by these changes. However, this may change as new therapeutic agents continue to be identified, such as for patients with sarcomas harbouring *NTRK* fusion genes [33], or in cases where melanoma has been misclassified as a MPNST [34].

Although the vast majority of genetic alterations were detected using the GEL variant calling pipelines, copy number alterations were not detected in *MDM2 and TBXT*, nor were *SSX2* fusion genes in synovial sarcoma identified. These alterations had been identified using FISH, and the *SSX2* fusion in particular was detected by visual inspection using Integrative Genomics Viewer [35]. This highlights that a rigorous validation processes is required when introducing any new tests into clinical practice. Improvement of the automated bioinformatic analysis pipeline and other tools will only be achieved with continued critical review.

### Remaining challenges and future perspectives

The 100KGP established that patients and healthcare workers alike are eager to engage in genomic testing, and that WGS can readily be incorporated into clinical service as a standard of care. Nevertheless, challenges remain. Obtaining sufficient fresh biopsy material will be necessary to deliver WGS consistently for sarcoma and other cancers. This represents a logistical challenge for non‐specialised NHS services that are not currently equipped with facilities such as liquid nitrogen and freezers. As sarcomas may arise anywhere in the body including in the breast, head and neck, retroperitoneum, and female genital tract, it is not uncommon for biopsies to be performed outside of specialist service centres. In these cases, obtaining a second sample may be required if WGS is deemed valuable. It also provides further support for early referral of patients to specialist centres, in line with the sarcoma service specification approved by NHS England in 2019 [36].

Going forward, delivery of WGS results will be aligned fully with the existing clinical pathway. From the point of patient entry into the service, obtaining consent and the processing of frozen tissue will be coordinated to eventually result in the generation of a WGS report. This report will be issued by the genomics team to the relevant clinicians and pathology department. Potentially actionable findings will be discussed at the sarcoma MDMs and relevant findings will be incorporated into the histopathology report. In practice, it is anticipated that only a minority of cases will require an in‐depth discussion akin to those currently held at our GTAB meetings. Based on our experience of the bimonthly National Ewing sarcoma MDMs [37], consideration is being given to establishing a national forum, with a panel of experts, to discuss challenging cases.

The long interval between taking a sample and interpreting the WGS results and issuing a report remains a challenge. Currently, tissue diagnoses using limited molecular techniques such as FISH are provided within 1 week, whereas delivery of WGS results takes approximately 6 weeks. With ongoing optimisation of the pathway, the turn‐around time could be reduced to as little as 2 weeks [38]. Until then, it will be necessary to continue to employ current standard of care diagnostic testing such as FISH, even when submitting samples for WGS. In light of these challenges, one might question whether it is right to pursue WGS as a standard of care test for patients with sarcoma. Would less‐complex methods such as targeted sequencing, RNA sequencing or methylation profiling resolve the challenges of sarcoma classification? Targeted sequencing is cheaper, with faster turn‐around times than for WGS. However, the information provided by targeted sequencing is limited, and has not been shown to provide significant patient benefit in an era where novel disease and therapeutic associations continue to be discovered.

Improvement of bioinformatics tools for detection of structural changes will address some of the challenges that were identified in this Project. Furthermore, the introduction of new sequencing technologies such as the Oxford Nanopore platform, which can provide long read sequences (>10 kb), will generate information permitting resolution of complex structural variants and enable inference of genome‐wide methylation profiles [39, 40]. WGS will be more cost‐effective as a result, with shorter turn‐around times than those offered by current technologies. However, delivery of a diagnostic service is a dynamic process which will change over time as we learn more of the underlying molecular alterations that underpin the pathogenesis of disease.

The most compelling reason to pursue WGS is to improve the survival of patients with sarcoma, something that has not happened in four decades [27]. The sarcoma data accrued from the 100KGP, combined with the WGS data obtained as part of standard of care testing in the future, will deliver an encyclopaedic catalogue of sarcoma genomics, made ever more powerful when linked dynamically to clinical outcome data. Generating other ‘omic’ data from the same samples will add value to the WGS. Such a compendium of data will be available indefinitely, and in time novel analytical methods will provide new insights into the pathogenesis of sarcoma [41, 42].

Finally, it must be recognised by NHS England that the introduction of genomic medicine as standard of care testing for sarcoma and other cancers will require additional resources. Additional staff will be required to discuss the impact of DNA sequencing with patients, particularly for parents of children with sarcoma, and to recruit patients into clinical trials. Additional time will be required for medical staff to consider new therapeutic options. Existing staff will need to take on new roles as some tasks become redundant. Investment in education in all spheres of healthcare delivery is required for the success of a modern medical service.

## Author contributions statement

AMF conceived the study. The study was designed by AMF, FA, SCP and ACS and each contributed equally to the writing of the manuscript. SCP and ACS performed data collection, analysis and interpretation, literature search and generation of figures. WC performed data analysis and interpretation. DL, AS, HT, RT, NP, JC and SSM performed data interpretation and assisted with writing of the manuscript. The RNOH Pathology Laboratory and Biobank Team consented all patients, and processed, quality controlled and tracked the tissue samples for sequencing. All authors were involved in final approval of the submitted manuscript.

## Supporting information


**Supplementary materials and methods**

**Table S1.** Summary of whole genome sequencing results from the first 350 patients whose results were analysed and discussed at the Genomic Tumour Advisory Board
