# Supplementary material for: Sarcoma and the 100,000 Genomes Project: our experience and changes to practice
Source: J Pathol Clin Res. 2020 Jun 23;6(4):297–307. doi: 10.1002/cjp2.174 (PMC7578291; doi:10.1002/cjp2.174)
Supplement: Supplementary file 1 — Supplementary materials and methods [file CJP2-6-297-s001.docx]

**Sarcoma and the 100,000 Genomes Project: our experience and changes to practice**

Prendergast SC *et al. J Pathol Clin Res* DOI: 10.1002/cjp2.174

The reference number refers to the main text reference list.

**Supplementary material and methods**

1. **A summary of the key steps for processing samples for RNA prior to extraction is outlined below.**

Samples were removed from the freezer and transferred on dry ice to the cryostat employing standard precautions to prevent contamination and RNA degradation. The frozen tumour tissue was mounted on a chuck using the embedding compound OCT. The frozen tissue block was trimmed. A section was then placed onto a glass slide, fixed with formalin, hand-stained with haematoxylin, and eosin, dehydrated and coverslipped. The section was then presented to a pathologist for review and scored using the template for assessment of the tumour quality according to GEL guidelines, as listed below*. Any focus contaminating non-neoplastic cells were marked by the pathologist on the slide, this was matched again the frozen block and cut out: a new section was then cut, stained and shown again to the pathologist who reassessed the quality of the material. If the sample failed the quality step significantly it was discarded

Once the tissue section passed the quality standards, 20-40 serial curls of tissue were cut into a 2ml safe-lock Eppendorf tube for DNA extraction, thereafter 6-10 curls were cut into a 2ml safe-lock Eppendorf tube containing 1ml of chilled TRIzol® reagent (Invitrogen™ # 15596026) and was immediately homogenised using a combination of pipetting and vortexing. The completely lysed sample was spun for 30 seconds at 8000rpm to remove undissolved debris. The supernatant containing the RNA was transferred into a 1.5ml RNAse-free Eppendorf tube and stored at -80C until RNA extraction was performed.

* 1)  Percentage tumour. The tissue section must be composed of at least 40% of the sample. *It is important to estimate this as the percentage of neoplastic vs non-neoplastic cells; e.g. if there is a prominent inflammatory cell infiltrate, the tumour percentage will be a lot lower. This is not simply the percentage of viable tumour on the slide which is a common misconception.*

2)       Percentage of tumour necrosis. Tumours showing 20% or more necrosis were excluded.

3)       Tumour cellularity. This is an approximate estimate of the number of tumour cells present on the section. The cut offs are as follows: Low cellularity: <4000 cells; moderate cellularity: 4,000 – 10,000 cells, high cellularity: 10,000-50,000 cells; very high cellularity: >50,000 cells.

1. **SPREC coding used for the samples [5]**

Type of tissue: TIS; BON

Type of collection: BPS; SRG

Long-term storage: C

Warm and cold ischemia: data not collected.

Type of Sample: BLD

Type of primary container: EDG; PXD

Long-term storage: J
