# Supplementary material for: Sarcoma and the 100,000 Genomes Project: our experience and changes to practice
Source: J Pathol Clin Res. 2020 Jun 23;6(4):297–307. doi: 10.1002/cjp2.174 (PMC7578291; doi:10.1002/cjp2.174)
Supplement: Supplementary file 2 — Table S1. Summary of whole genome sequencing results from the first 350 patients whose results were analysed and discussed at the Genomic Tumour Advisory Board [file CJP2-6-297-s002.docx]

**Sarcoma and the 100,000 Genomes Project: our experience and changes to practice**

Prendergast SC *et al. J Pathol Clin Res* DOI: 10.1002/cjp2.174

**Table S1.** Summary of whole genome sequencing results from the first 350 patients whose results were analysed and discussed at the Genomic Tumour Advisory Board

| **Study ID** | **Age at diagnosis** | **Histology Main Diagnosis** | **Histology Subtype Diagnosis** | **Modified Diagnosis after GTAB** | **Gender** | **Primary/LR/Metastatic sample WGS** | **Primary in Bone or Soft Tissue** | **Tumour Grade** | **Domain 1 (n) and diagnostic relevant Domain 2*** | **Tier 1 and disease relevant ^£^** |
| --- | --- | --- | --- | --- | --- | --- | --- | --- | --- | --- |
| 1 | 41 | Osteosarcoma | Giant cell rich | No | M | P | Bone | 3 | TP53, RB1 | not detected |
| 2 | 43 | Chondrosarcoma | Conventional central | No | M | P | Bone | 2 | IDH1 | not detected |
| 3 | 70 | Myxofibrosarcoma | NA | No | F | P | Soft Tissue | 2 | Not detected | not detected |
| 4 | 67 | Myxofibrosarcoma | NA | No | F | P | Soft Tissue | 2 | Not detected | not detected |
| 5 | 53 | Osteosarcoma | Osteoblastic and chondroblastic | No | M | P | Bone | 3 | Not detected | not detected |
| 6 | 67 | Chordoma | Conventional | No | M | P | Bone | 1 | Not detected | not detected |
| 7 | 64 | Leiomyosarcoma | Pleomorphic | No | M | P | Soft Tissue | 3 | NF1, MAP2K1 | not detected |
| 8 | 18 | Ewing sarcoma | NA | No | F | Met | Soft Tissue | 4 | Not detected | not detected |
| 9 | 56 | Chondrosarcoma | Conventional central | No | M | P | Bone | 2 | IDH1 | not detected |
| 10 | 68 | Synovial sarcoma | Biphasic | No | F | P | Soft Tissue | 2 | TSC1 | not detected |
| 11 | 68 | Adamantinoma | NA | No | M | LR | Bone | 3 | Not detected | not detected |
| 12 | 52 | Myxofibrosarcoma | NA | No | M | P | Soft Tissue | 2 | CTNNA2 | not detected |
| 13 | 12 | Osteosarcoma | Chondroblastic | No | M | LR | Bone | 3 | MCPH1 | not detected |
| 14 | 12 | Ewing sarcoma | NA | No | F | P | Bone | 4 | Not detected | not detected |
| 15 | 27 | Chondrosarcoma | Conventional central | No | F | P | Bone | 1 | IDH1 | not detected |
| 16 | 74 | Myxofibrosarcoma | NA | No | F | P | Soft Tissue | 3 | Not detected | not detected |
| 17 | 68 | Chondrosarcoma | Conventional central | No | F | P | Bone | 2 | IDH1, CTNNA1 | not detected |
| 18 | 77 | Myxofibrosarcoma | NA | No | M | P | Soft Tissue | 1 | PIK3R2 | SDHA^£^ |
| 19 | 49 | Myxofibrosarcoma | NA | No | F | P | Soft Tissue | 3 | Not detected | not detected |
| 20 | 50 | Clear cell sarcoma | NA | No | M | Met | Soft Tissue | 3 | Not detected | not detected |
| 21 | 75 | Myxofibrosarcoma | NA | No | M | P | Soft Tissue | 3 | FGF3 , FGFR3 | not detected |
| 22 | 62 | Leiomyosarcoma | Conventional | No | F | P | Soft Tissue | 3 | ATRX, RB1 | TP53 |
| 23 | 49 | Synovial sarcoma | Monophasic fibrous type | No | M (previously F) | P | Soft Tissue | 2 | Not detected | not detected |
| 24 | 38 | Osteosarcoma | Chondroblastic and osteoblastoma-like areas | No | M | P | Bone | 3 | FANCD2, STAG2 | not detected |
| 25 | 55 | Chondrosarcoma | Conventional central | No | F | P | Bone | 2 | IDH1, TP53 | not detected |
| 26 | 32 | Chondrosarcoma | Conventional secondary peripheral | No | M | LR | Bone | 2 | CHEK2, MSH2 | not detected |
| 27 | 67 | Spindle cell sarcoma | NA | No | M | P | Soft Tissue | 3 | EGFR | not detected |
| 28 | 35 | Chondrosarcoma | Conventional peripheral | No | F | LR | Bone | 1 | Not detected | not detected |
| 29 | 78 | Leiomyosarcoma | Conventional | No | M | P | Bone | 3 | ROS1, TP53 | not detected |
| 30 | 70 | Myxofibrosarcoma | NA | No | M | Met | Soft Tissue | 3 | MSH6 | not detected |
| 31 | 65 | MPNST | Conventional | No | M | P | Soft Tissue | 3 | Not detected | not detected |
| 32 | 72 | Chondrosarcoma | Conventional central | No | F | LR | Bone | 3 | IDH1, TSC1 | not detected |
| 33 | 73 | Myxofibrosarcoma | NA | No | F | P | Soft Tissue | 2 | Not detected | not detected |
| 34 | 53 | Leiomyosarcoma | Conventional | No | F | Met | Soft Tissue | 3 | ATRX, TP53, RNF43 | not detected |
| 35 | 69 | Myxofibrosarcoma | NA | No | M | P | Soft Tissue | 3 | ATRX, RB1 | not detected |
| 36 | 59 | Spindle cell sarcoma | NA | No | F | LR | Soft Tissue | 2 | TP53 | not detected |
| 37 | 24 | Chordoma | Conventional | No | M | P | Bone | 2 | Not detected | not detected |
| 38 | 83 | Leiomyosarcoma | Conventional | No | F | P | Soft Tissue | 3 | PDGFRA, RB1 (2), TP53 | not detected |
| 39 | 28 | Chondrosarcoma | Periosteal | No | M | P | Bone | 2 | Not detected | not detected |
| 40 | 48 | Chondrosarcoma | Conventional central | No | M | P | Bone | 2 | Not detected | not detected |
| 41 | 50 | Adamantinoma | Osteofibrous-dysplasia-like | No | F | P | Bone | 1 | Not detected | not detected |
| 42 | 77 | Myxofibrosarcoma | NA | No | M | P | Soft Tissue | 3 | Not detected | not detected |
| 43 | 71 | Spindle cell sarcoma | NA | No | M | P | Bone | 3 | TP53 | not detected |
| 44 | 28 | Clear cell sarcoma | NA | No | F | P | Soft Tissue | 3 | FANCE | not detected |
| 45 | 64 | Spindle cell sarcoma | NA | No | F | P | Bone | 3 | BRIP1 | not detected |
| 46 | 63 | Chordoma | Conventional | No | M | P | Bone | 2 | Not detected | not detected |
| 47 | 56 | Leiomyosarcoma | Conventional | No | F | Met | Soft Tissue | 3 | Not detected | not detected |
| 48 | 78 | Myxofibrosarcoma | NA | No | M | P | Soft Tissue | 2 | EMSY, NF1 | not detected |
| 49 | 26 | Epithelioid sarcoma | Classic | No | F | P | Soft Tissue | 3 | FANCD2 | not detected |
| 50 | 41 | Alveolar soft part sarcoma | NA | No | M | P | Soft Tissue | 3 | Not detected | not detected |
| 51 | 81 | Leiomyosarcoma | Conventional | No | F | P | Soft Tissue | 3 | Not detected | not detected |
| 52 | 48 | Chondrosarcoma | Dedifferentiated central | No | F | P | Bone | 3 | ATRX | not detected |
| 53 | 63 | Pleomorphic sarcoma | Unusual osteoclast-rich | No | F | P | Soft Tissue | 3 | Not detected | not detected |
| 54 | 36 | Liposarcoma | Pleomorphic | No | M | P | Soft Tissue | 3 | PIK3CG | not detected |
| 55 | 56 | Osteosarcoma | Osteoblastic | No | M | P | Bone | 3 | Not detected | not detected |
| 56 | 25 | Sclerosing epithelioid fibrosarcoma | NA | No | F | P | Bone | 3 | Not detected | not detected |
| 57 | 77 | Myxofibrosarcoma | NA | No | F | P | Soft Tissue | 3 | NF1 | not detected |
| 58 | 9 | Osteosarcoma | Chondroblastic | No | M | P | Bone | 3 | ATRX | not detected |
| 59 | 71 | Myxofibrosarcoma | NA | No | M | P | Soft Tissue | 3 | CTNNA2 | not detected |
| 60 | 61 | Spindle cell sarcoma | NA | No | M | P | Bone | 3 | Not detected | not detected |
| 61 | 57 | Chordoma | Conventional | No | M | P | Bone | NA | Not detected | not detected |
| 62 | 48 | Chordoma | Poorly differentiated | No | M | P | Bone | NA | Not detected | not detected |
| 63 | 63 | Chondrosarcoma | Dedifferentiated central | No | M | P | Bone | 3 | NF1, TP53, IDH2* | not detected |
| 64 | 67 | Chondrosarcoma | Conventional central | No | F | P | Bone | 2 | Not detected | not detected |
| 65 | 65 | Solitary fibrous tumour | NA | No | M | P | Soft Tissue | NA | Not detected | not detected |
| 66 | 26 | Osteosarcoma | Parosteal | No | F | P | Bone | 1 | Not detected | not detected |
| 67 | 33 | Chondrosarcoma | Conventional secondary peripheral | No | M | P | Bone | 1 | TP53 | not detected |
| 68 | 61 | Chordoma | Conventional | No | F | Met | Bone | NA | PTEN | not detected |
| 69 | 65 | Chordoma | Conventional | No | F | LR | Bone | 2 | CTNNA3 | not detected |
| 70 | 62 | Myxofibrosarcoma | NA | No | F | P | Soft Tissue | 2 | Not detected | not detected |
| 71 | 80 | Leiomyosarcoma | Conventional | No | F | P | Soft Tissue | 2 | TP53, RB1 | not detected |
| 72 | 67 | Osteosarcoma | Soft Tissue | No | M | P | Soft Tissue | 3 | ATRX | not detected |
| 73 | 54 | Chondrosarcoma | Conventional central | No | M | P | Bone | 2 | IDH1 | not detected |
| 74 | 63 | Chondrosarcoma | Conventional central | No | F | LR | Bone | 2 | IDH2* GNAS* | not detected |
| 75 | 84 | Pleomorphic sarcoma | Undifferentiated | No | M | P | Bone | 3 | BRCA2 (3), MSH2, TP53 (5), NF1, TSC1, TSC2 | not detected |
| 76 | 28 | Chordoma | Conventional | No | M | P | Bone | 2 | Not detected | not detected |
| 77 | 51 | Chondrosarcoma | Conventional central | No | M | P | Bone | 2 | IDH1 | not detected |
| 78 | 66 | Sclerosing epithelioid fibrosarcoma | NA | No | M | P | Soft Tissue | 3 | CTNNA2 | not detected |
| 79 | 16 | Osteoclast-rich sarcoma | Radiation-induced (secondary malignant GCT) | No | F | P | Bone | NA | Not detected | TP53 |
| 80 | 78 | Pleomorphic sarcoma | Undifferentiated | No | M | P | Soft Tissue | 3 | FANCM | not detected |
| 81 | 30 | Chondrosarcoma | Mesenchymal | No | F | P | Soft Tissue | 3 | ALK | not detected |
| 82 | 36 | Synovial sarcoma | Monophasic fibrous type | No | M | P | Soft Tissue | 2 | IDH1 | not detected |
| 83 | 66 | Myxofibrosarcoma | NA | No | M | P | Soft Tissue | 2 | Not detected | not detected |
| 84 | 49 | Spindle cell sarcoma | Osteosarcomatous differentiation | No | M | P | Soft Tissue | 3 | Not detected | not detected |
| 85 | 50 | Osteosarcoma | Fibroblastic | No | M | P | Bone | 3 | FGF20 | not detected |
| 86 | 48 | Ewing sarcoma | NA | No | F | P | Bone | 4 | Not detected | not detected |
| 87 | 57 | Leiomyosarcoma | Pleomorphic | No | F | Met | Soft Tissue | 3 | Not detected | not detected |
| 88 | 58 | Myxofibrosarcoma | NA | No | M | LR | Soft Tissue | 3 | Not detected | not detected |
| 89 | 25 | Chondrosarcoma | Mesenchymal | No | F | Met | Bone | 3 | Not detected | not detected |
| 90 | 81 | Pleomorphic sarcoma | Undifferentiated - osteoclast-rich | No | M | P | Soft Tissue | 3 | FGF14, RB1, ATRX | not detected |
| 91 | 28 | Spindle cell sarcoma | Myofibroblastic differentiation | No | M | Met | Soft Tissue | 2 | Not detected | not detected |
| 92 | 78 | Chondrosarcoma | Conventional central | No | M | P | Bone | 2 | CDKN2A, IDH2* | not detected |
| 93 | 67 | Chondrosarcoma | Conventional central | No | M | LR | Bone | 2 (with focal transition to G3) | IDH2* | not detected |
| 94 | 50 | Leiomyosarcoma | NA | No | F | P | Soft Tissue | 2 | RB1 | not detected |
| 95 | 21 | Epithelioid sarcoma | Classic | No | F | P | Soft Tissue | 3 | SMARCB1 | not detected |
| 96 | 81 | Myxofibrosarcoma | NA | No | M | P | Soft Tissue | 2 | PALB2, ATRX, RICTOR | TP53 |
| 97 | 57 | Leiomyosarcoma | Pleomorphic | No | F | P | Soft Tissue | 3 | Not detected | not detected |
| 98 | 31 | Osteosarcoma | Telangiectatic | No | M | LR/Met | Bone | 3 | FGF19 | not detected |
| 99 | 55 | Chondrosarcoma | Conventional central | No | M | P | Bone | 3 | IDH1, RNF43 | not detected |
| 100 | 61 | Chondrosarcoma | Conventional central | No | F | LR | Bone | 1 | IDH1, CDKN2A | not detected |
| 101 | 57 | Malignant solitary fibrous tumour | NA | No | M | Met | Soft Tissue | 3 | ROS1 | not detected |
| 102 | 35 | MPNST | Rhabdomyoblastic differentiation - malignant Triton | No | M | LR/Met | Soft Tissue | 3 | TP53 | not detected |
| 103 | 27 | Alveolar soft part sarcoma | NA | No | F | P | Soft Tissue | 3 | Not detected | not detected |
| 104 | 22 | Chondrosarcoma | Conventional central | No | F | LR | Bone | 1 | Not detected | not detected |
| 105 | 18 | Alveolar soft part sarcoma | NA | No | F | P | Soft Tissue | 3 | Not detected | not detected |
| 106 | 26 | Chondrosarcoma | Mesenchymal | No | M | P | Soft Tissue | 3 | Not detected | not detected |
| 107 | 41 | Synovial sarcoma | Monophasic fibrous type | No | M | P | Soft Tissue | 2 | Not detected | not detected |
| 108 | 32 | Adamantinoma | NA | No | M | Met | Bone | NA | Not detected | not detected |
| 109 | 64 | Spindle cell sarcoma | NA | No | F | P | Soft Tissue | 2 | Not detected | not detected |
| 110 | 77 | Myxofibrosarcoma | NA | No | M | P | Soft Tissue | 3 | AKT1 | not detected |
| 111 | 21 | Chondrosarcoma | Clear cell | No | M | LR | Bone | 2 | Not detected | not detected |
| 112 | 30 | Chondrosarcoma | Conventional central | No | M | P | Bone | 2 | Not detected | not detected |
| 113 | 31 | Chondrosarcoma | Mesenchymal | No | F | P | Bone | 3 | ARAF | not detected |
| 114 | 78 | Leiomyosarcoma | Pleomorphic | No | M | P | Soft Tissue | 3 | TP53 | not detected |
| 115 | 37 | Liposarcoma | Myxoid | No | M | P | Soft Tissue | 3 | ATM | not detected |
| 116 | 28 | Chondrosarcoma | Conventional central | No | M | P | Bone | 2 | Not detected | not detected |
| 117 | 31 | Clear cell sarcoma | NA | No | M | P | Soft Tissue | 3 | Not detected | not detected |
| 118 | 78 | Pleomorphic sarcoma | Undifferentiated | No | F | Met | Soft Tissue | 3 | TP53, NTRK1, ALK | not detected |
| 119 | 34 | Clear cell sarcoma | NA | No | F | LR | Soft Tissue | 3 | Not detected | not detected |
| 120 | 56 | Liposarcoma | Myxoid | No | M | P | Soft Tissue | 3 | NBN | not detected |
| 121 | 77 | Myxofibrosarcoma | NA | No | F | P | Soft Tissue | 3 | FGF4, TP53 | not detected |
| 122 | 46 | Leiomyosarcoma | Pleomorphic | No | M | P | Soft Tissue | 3 | Not detected | not detected |
| 123 | 21 | Osteosarcoma | Difficult to subtype | No | M | P | Bone | 3 | TP53 | not detected |
| 124 | 47 | Chondrosarcoma | Dedifferentiated central | No | F | P | Bone | 3 | IDH1, TP53 | not detected |
| 125 | 54 | Pleomorphic sarcoma | Undifferentiated | No | M | P | Soft Tissue | 3 | FGF13 | not detected |
| 126 | 31 | Synovial sarcoma | Monophasic fibrous type | No | M | P | Soft Tissue | 2 | Not detected | not detected |
| 127 | 43 | Leiomyosarcoma | Epithelioid | No | F | P | Bone | 3 | KLB | not detected |
| 128 | 90 | Myxofibrosarcoma | NA | No | M | P | Soft Tissue | 2 | TP53, FGFR4, MYC | not detected |
| 129 | 12 | Osteosarcoma | Osteoblastic | No | F | P | Bone | 3 | Not detected | TP53 |
| 130 | 62 | Malignant Solitary Fibrous Tumour | NA | No | F | Met | Soft Tissue | 3 | TP53 | not detected |
| 131 | 53 | Chondrosarcoma | Conventional central | No | F | P | Bone | 1 | IDH1 | not detected |
| 132 | 14 | Osteosarcoma | Osteoblastic and chondroblastic | No | M | LR | Bone | 3 | Not detected | not detected |
| 133 | 53 | Leiomyosarcoma | Conventional | No | F | P | Soft Tissue | 2 | TP53, MAPK1 | not detected |
| 134 | 40 | Chondrosarcoma | Mesenchymal | No | M | P | Bone | 3 | Not detected | not detected |
| 135 | 74 | Myxofibrosarcoma | NA | No | M | P | Soft Tissue | 2 on bx, 3 on resection | Not detected | not detected |
| 136 | 50 | Myxofibrosarcoma | NA | No | M | LR | Soft Tissue | 3 | ATRX, ROS1, KLB | not detected |
| 137 | 66 | Spindle cell sarcoma | High grade | No | M | P | Soft Tissue | 3 | RICTOR | not detected |
| 138 | 70 | Chondrosarcoma | Conventional central | No | F | P | Bone | 2 | IDH2 * | not detected |
| 139 | 77 | Pleomorphic sarcoma | Undifferentiated | No | M | LR | Soft Tissue (with bone invasion - femur) | 3 | Not detected | not detected |
| 140 | 79 | Leiomyosarcoma | Conventional | No | F | P | Soft Tissue | 2 | Not detected | not detected |
| 141 | 56 | Myxofibrosarcoma | NA | No | M | P | Soft Tissue | 3 | ATRX | not detected |
| 142 | 25 | Osteosarcoma | Parosteal | No | F | P | Bone | 1 | ATRX, NF1 | not detected |
| 143 | 35 | Osteosarcoma | Central | No | M | P | Bone | 3 | Not detected | not detected |
| 144 | 67 | Leiomyosarcoma | Cutaneous | No | M | P | Soft Tissue | 2 | ATRX, TP53 (2), FGF19, RB1 | not detected |
| 145 | 42 | Angiosarcoma | Epithelioid | No | M | P | Soft Tissue | 3 | Not detected | not detected |
| 146 | 77 | Myxofibrosarcoma | NA | No | M | P | Soft Tissue | 3 | FRS2 | not detected |
| 147 | 53 | Myxofibrosarcoma | NA | No | M | LR | Soft Tissue | 2 | FANCD2, CTNNA3, FGF20 | not detected |
| 148 | 76 | Liposarcoma | Dedifferentiated | No | M | LR | Soft Tissue | 3 | Not detected | not detected |
| 149 | 85 | Liposarcoma | Pleomorphic | No | F | P | Soft Tissue | 3 | TP53, RB1, FANCD2 | not detected |
| 150 | 20 | Osteosarcoma | Parosteal | No | M | P | Bone | 1 | Not detected | not detected |
| 151 | 34 | Liposarcoma | Pleomorphic | No | F | P | Soft Tissue | 3 | Not detected | not detected |
| 152 | 26 | Chondrosarcoma | Extraskeletal myxoid | No | M | P | Soft Tissue | 2 | Not detected | not detected |
| 153 | 67 | Synovial sarcoma | Biphasic | No | F | LR | Soft Tissue | 2 | CTNNB1 , | not detected |
| 154 | 77 | Myxofibrosarcoma | NA | No | M | LR | Soft Tissue | 2 | FGFR1, PARP1, AKT2, SLX4 | not detected |
| 155 | 30 | Myofibrosarcoma | NA | No | M | P | Bone | 3 | RB1 | TP53 |
| 156 | 15 | Synovial sarcoma | Monophasic fibrous type | No | F | LR | Soft Tissue | 2 | STAG2 | not detected |
| 157 | 63 | Chondrosarcoma | Extraskeletal myxoid | No | M | P | Soft Tissue | 3 | Not detected | not detected |
| 158 | 62 | Myxofibrosarcoma | NA | No | F | P | Soft Tissue | 2 | STAG2 | not detected |
| 159 | 62 | Spindle cell sarcoma | NA | No | M | P | Soft Tissue | 2 | ATRX, PIK3R1 | not detected |
| 160 | 34 | Spindle cell sarcoma | Myofibroblastic differentiation | No | F | LR | Soft Tissue | 3 | ATRX, RB1, TP53 | not detected |
| 161 | 68 | Myxofibrosarcoma | NA | No | M | P | Soft Tissue | 3 | Not detected | not detected |
| 162 | 41 | Leiomyosarcoma | Conventional | No | M | P, Met | Soft Tissue, Bone | 3 | CTNNA, TP53 | not detected |
| 163 | 57 | Spindle cell sarcoma | Osteosarcomatous differentiation | No | F | LR | Soft Tissue | 3 | BRCA2, PDGFRA, TP53 | not detected |
| 164 | 54 | Chondrosarcoma | Conventional secondary peripheral | Chondrosarcoma conventional central/periosteal | F | P | Bone | 2 | IDH1 | not detected |
| 165 | 63 | Chondrosarcoma | Conventional secondary peripheral | Chondrosarcoma conventional central/periosteal | M | P | Bone | 2 | IDH1, PIK3CA | not detected |
| 166 | 79 | Pleomorphic sarcoma | Undifferentiated | No | F | P | Soft Tissue | 3 | ATRX, NF1 | not detected |
| 167 | 43 | Chordoma | Conventional | No | F | LR | Bone | NA | KLB | not detected |
| 168 | 81 | Myxofibrosarcoma | NA | No | F | P | Soft Tissue | 3 | Not detected | not detected |
| 169 | 13 | Osteosarcoma | Chondroblastic | No | F | LR | Bone | 3 | MYC, RICTOR, TSC1 | not detected |
| 170 | 68 | High grade sarcoma with osteosarcomatous differentiation | Possibly radiation-induced | No | F | P | Bone | 3 | SMARCA4, ROS1, TSC1 | not detected |
| 171 | 37 | Pleomorphic sarcoma | Undifferentiated | No | M | P | Soft Tissue | 3 | FANCD2 | not detected |
| 172 | 12 | Osteosarcoma | Osteoblastic | No | F | P | Bone | 3 | MSH2, TP53 | not detected |
| 173 | 74 | Osteosarcoma | Osteoblastic | No | F | P | Bone | 3 | TP53 (3), FANCD2 | not detected |
| 174 | 38 | Malignant GCT | Conventional | No | F | P | Bone | 3 | Not detected | not detected |
| 175 | 25 | Epithelioid sarcoma | Proximal | No | F | P | Soft Tissue | NA | ARAF | not detected |
| 176 | 45 | Chondrosarcoma | Conventional central | No | F | LR | Bone | 2 | IDH1, KLB, CDKN2A | not detected |
| 177 | 13 | Chondrosarcoma | Mesenchymal | No | F | P | Soft Tissue | 3 | Not detected | not detected |
| 178 | 28 | Chordoma | Extra-axial soft tissue | No | F | P | Soft Tissue | NA | Not detected | not detected |
| 179 | 69 | Chordoma | Conventional | No | F | P | Bone | NA | Not detected | not detected |
| 180 | 30 | Chondrosarcoma | Conventional central | No | M | P | Bone | 1 | IDH1 | not detected |
| 181 | 26 | Chordoma | Conventional | No | F | P | Bone | NA | Not detected | not detected |
| 182 | 65 | Chondrosarcoma | Conventional central | No | F | P | Bone | 2 | PMS1, IDH1 | not detected |
| 183 | 83 | Liposarcoma | Pleomorphic | No | F | P | Soft Tissue | 3 | FGF12 | not detected |
| 184 | 47 | Chondrosarcoma | Conventional central | No | M | LR | Bone | 2 | Not detected | not detected |
| 185 | 74 | Chondrosarcoma | Conventional central | No | F | P | Bone | 2 | IDH2 * | not detected |
| 186 | 26 | Liposarcoma | pleomorphic | Undifferentiated pleomorphic sarcoma (PRDM10-MED12 rearranged sarcoma) | M | P | Soft Tissue | 3 | Not detected | not detected |
| 187 | 51 | Liposarcoma | Unusual pleomorphic | No | M | P | Soft Tissue | 1 | NF1, PTEN (3), TP53 (2), TSC2 | not detected |
| 188 | 12 | Osteosarcoma | Osteoblastic | No | F | P | Bone | 3 | FGF16, CTNN3A | not detected |
| 189 | 61 | Osteosarcoma | Telangiectatic | No | F | P | Bone | 3 | RB1, FGF10 | not detected |
| 190 | 68 | Osteosarcoma | Radiation-induced | No | F | P | Bone | 3 | ATM, RICTOR | not detected |
| 191 | 37 | Osteosarcoma | Soft Tissue | No | M | P | Soft Tissue | 3 | AKT2 | not detected |
| 192 | 11 | Osteosarcoma | Telangiectatic | No | F | P | Bone | 3 | Not detected | not detected |
| 193 | 16 | Osteosarcoma | Mixed osteoblastic and telangiectatic-type | No | F | P | Bone | 3 | RB1 | not detected |
| 194 | 17 | Malignant GCT | Low grade | No | F | P | Bone | 1 | Not detected | not detected |
| 195 | 72 | Epithelioid tumour | Epithelioid MPNST vs malignant melanoma | Melanoma | M | P | Soft Tissue | NA | BRCA2, CTNNA2, CTNNA3, EGFR, FANCI, FANCM, FGF10, FGF12 (2), MET, ROS1 | not detected |
| 196 | 54 | Low grade fibromyxoid sarcoma | NA | No | M | P | Soft Tissue | 1 | Not detected | not detected |
| 197 | 24 | Synovial sarcoma | Poorly differentiated | No | M | LR | Soft Tissue | 3 | PDGFRA | not detected |
| 198 | 26 | Osteosarcoma | Parosteal | No | F | P | Bone | 1 | Not detected | not detected |
| 199 | 60 | Synovial sarcoma | Monophasic fibrous type | No | F | P | Soft Tissue | 2 | Not detected | not detected |
| 200 | 9 | Synovial sarcoma | Biphasic | No | M | P | Soft Tissue | 2 | ROS1 | not detected |
| 201 | 9 | Rhabdomyosarcoma | Alveolar | No | F | P | Soft Tissue | 3 | Not detected | not detected |
| 202 | 76 | Myxofibrosarcoma | NA | No | M | LR | Soft Tissue | 3 | FANCD2, MLH3 | not detected |
| 203 | 77 | Liposarcoma | Dedifferentiated | No | M | P | Soft Tissue | 2 | Not detected | not detected |
| 204 | 61 | Liposarcoma | Pleomorphic | No | M | P | Soft Tissue | 3 | ATRX, TP53, PIK3CA | not detected |
| 205 | 25 | Ewing sarcoma | NA | No | M | P | Bone | 4 | Not detected | not detected |
| 206 | 80 | Myxofibrosarcoma | NA | No | M | P | Soft Tissue | 3 | RNF43, TP53 | not detected |
| 207 | 17 | Malignant mixed tumour | NA | No | M | P | Soft Tissue | 3 | Not detected | not detected |
| 208 | 70 | Rhabdomyosarcoma | Pleomorphic | No | M | P | Soft Tissue | 3 | Not detected | not detected |
| 209 | 75 | Leiomyosarcoma | Conventional | No | F | P | Soft Tissue | 3 | Not detected | not detected |
| 210 | 51 | Chondrosarcoma | Extraskeletal myxoid | No | M | Met | Soft Tissue | 3 | NRAS | not detected |
| 211 | 37 | Ewing sarcoma | NA | No | M | P | Bone | 4 | Not detected | not detected |
| 212 | 37 | Spindle cell sarcoma | NA | No | F | P | Soft Tissue | NA | Not detected | not detected |
| 213 | 49 | Rhabdomyosarcoma | Pleomorphic | No | M | P | Soft Tissue | 3 | FGF1, TP53 | not detected |
| 214 | 36 | Osteosarcoma | Arising in fibrous dysplasia | No | F | P | Bone | 3 | BRIP1, RICTOR, TP53, GNAS * | not detected |
| 215 | 54 | Spindle cell sarcoma | Myofibroblastic differentiation | No | F | P | Bone | 3 | Not detected | not detected |
| 216 | 45 | Clear cell sarcoma | NA | No | F | P | Soft Tissue | 3 | NF1, MET | not detected |
| 217 | 5 | Ewing sarcoma | NA | No | M | P | Bone | 4 | PIK3CG | not detected |
| 218 | 58 | Chondrosarcoma | Conventional central | No | M | P | Bone | 2 | BRCA2, FANCG, FGFR4, MRE11, MSH2, PARP1 | not detected |
| 219 | 27 | Malignant GCT | Conventional | No | F | P | Bone | 3 | Not detected | not detected |
| 220 | 51 | Myxofibrosarcoma | NA | No | F | LR | Soft Tissue | 2 | TP53 | not detected |
| 221 | 38 | Leiomyosarcoma | Conventional | No | M | P | Soft Tissue | 1 | FANCB, MAPK1 | not detected |
| 222 | 50 | MPNST | Conventional | Dermatofibrosarcoma protuberans | M | P | Soft Tissue | 3 | Not detected | not detected |
| 223 | 67 | Myxofibrosarcoma | NA | No | M | P | Soft Tissue | 2 | FANCM | not detected |
| 224 | 68 | Synovial sarcoma | Monophasic fibrous type | No | M | P | Soft Tissue | 2 | Not detected | not detected |
| 225 | 12 | Osteosarcoma | Chondroblastic | No | F | P | Bone | 3 | RB1 | not detected |
| 226 | 12 | Osteosarcoma | Osteoblastoma-like | No | F | P | Bone | 1 | ATRX | not detected |
| 227 | 57 | Leiomyosarcoma | Conventional | No | M | P | Soft Tissue | 3 | Not detected | not detected |
| 228 | 70 | Liposarcoma | Dedifferentiated | No | F | P | Soft Tissue | 3 | Not detected | not detected |
| 229 | 15 | Low grade fibromyxoid sarcoma | NA | No | F | P | Soft Tissue | 1 | Not detected | not detected |
| 230 | 46 | Leiomyosarcoma | Conventional | No | M | P | Soft Tissue | 2 | FANCD2 | not detected |
| 231 | 22 | Chondrosarcoma | Conventional central | No | M | P | Bone | 1 | IDH1, PIK3CA | not detected |
| 232 | 5 | Ewing sarcoma | NA | No | M | P | Bone | 4 | Not detected | not detected |
| 233 | 39 | Pleomorphic sarcoma | Undifferentiated | No | M | P | Soft Tissue | 3 | RB1, TP53 | not detected |
| 234 | 80 | Myxofibrosarcoma | NA | No | F | P | Soft Tissue | 3 | TP53 | not detected |
| 235 | 21 | Ewing sarcoma | NA | No | M | Met | Soft Tissue | 4 | EZH12 | not detected |
| 236 | 13 | Angiomatoid fibrous histiocytoma | NA | No | F | P | Soft Tissue | 1 | Not detected | not detected |
| 237 | 21 | Synovial sarcoma | Monophasic fibrous type | No | F | P | Soft Tissue | 2 | Not detected | not detected |
| 238 | 51 | Leiomyosarcoma | Conventional | No | F | P | Soft Tissue | 3 | Not detected | not detected |
| 239 | 74 | Chondrosarcoma | Conventional central | No | M | P | Bone | 1 | Not detected | not detected |
| 240 | 74 | Myxofibrosarcoma | NA | No | M | Met | Soft Tissue | 3 | NF1 , SLX4 | not detected |
| 241 | 36 | Myxofibrosarcoma | NA | No | F | P | Soft Tissue | 2 | MLH1, TP53 | not detected |
| 242 | 39 | Chondrosarcoma | Conventional central | No | M | P | Bone | 2 | IDH2 * | not detected |
| 243 | 25 | Epithelioid sarcoma | Classic | No | F | P | Soft Tissue (with bone invasion - scapula) | 3 | BRCA1 | not detected |
| 244 | 82 | Pleomorphic sarcoma | Undifferentiated | No | M | P | Soft Tissue | 2 | FGFR2 | not detected |
| 245 | 44 | Osteosarcoma | High grade parosteal | No | F | P | Bone | 3 | Not detected | not detected |
| 246 | 70 | Chondrosarcoma | Dedifferentiated central | No | M | P | Bone | 3 | IDH1 | not detected |
| 247 | 15 | Osteosarcoma | Osteoblastic | No | M | P, Met | Bone | 3 | Not detected | not detected |
| 248 | 45 | Chordoma | Conventional | No | M | LR | Bone | 2 | NF1 | not detected |
| 249 | 12 | Osteosarcoma | Periosteal | No | F | P | Bone | 3 | TP53 | not detected |
| 250 | 9 | Osteosarcoma | Osteoblastic | No | F | P | Bone | 3 | Not detected | TSC2 |
| 251 | 45 | Dermatofibrosarcoma protuberans | Fibrosarcomatous transformation | No | M | P | Soft Tissue | 2 | NF1, XRCC1 | not detected |
| 252 | 9 | Ewing sarcoma | NA | No | M | P | Bone | NA | Not detected | not detected |
| 253 | 82 | Myxofibrosarcoma | NA | No | F | P | Soft Tissue | 2 | TP53 | not detected |
| 254 | 39 | Synovial sarcoma | Biphasic | No | F | P | Soft Tissue | 2 | Not detected | not detected |
| 255 | 55 | Myxofibrosarcoma | NA | No | F | P | Soft Tissue | 3 | ATRX (2), FANCD2 | not detected |
| 256 | 7 | Low grade fibromyxoid sarcoma | NA | No | M | P | Soft Tissue | 1 | Not detected | not detected |
| 257 | 30 | Spindle cell sarcoma | High grade | No | M | P | Soft Tissue | 3 | Not detected | not detected |
| 258 | 40 | Chondrosarcoma | Conventional central | No | M | P | Bone | 2 | Not detected | not detected |
| 259 | 31 | Myxofibrosarcoma | NA | No | M | P | Soft Tissue | 3 | BRIP1, CTNN2A, MSH6, NF1, PPP2RA, PTEN, RAF1, TP53 | not detected |
| 260 | 17 | Osteosarcoma | Chondroblastic | No | F | P | Bone | 3 | Not detected | not detected |
| 261 | 79 | Chondrosarcoma | Dedifferentiated central | No | M | P | Bone | 2 | IDH1, TP53, CDKN2A (2) | not detected |
| 262 | 29 | Low grade fibromyxoid sarcoma | NA | No | M | P | Soft Tissue | NA | SLX4 | not detected |
| 263 | 68 | Liposarcoma | Dedifferentiated | No | M | P | Soft Tissue | 3 | FANCD2 | not detected |
| 264 | 36 | Spindle cell sarcoma | Radiation-induced | No | F | P | Soft Tissue | 3 | CTNNB1, TP53, TSC1, MSH6 | not detected |
| 265 | 10 | Osteosarcoma | Central low grade | No | M | P | Bone | 1 | Not detected | SQSTM1 |
| 266 | 43 | Sarcoma - unusual | Features favour an atypical ossifying fibromyxoid tumour | No | F | P | Soft Tissue | 1 | MLH1, CTNNB1 | not detected |
| 267 | 24 | Liposarcoma | Myxoid | No | M | P | Soft Tissue | 2 | MET | not detected |
| 268 | 71 | Liposarcoma | Dedifferentiated with osteosarcomatous differentiation | No | M | P | Soft Tissue | 3 | BAP1, FGF5, MAP3K1 | not detected |
| 269 | 22 | Spindle cell sarcoma | NA | No | F | P | Bone | 3 | Not detected | not detected |
| 270 | 12 | Ewing sarcoma | NA | No | M | P | Bone | 4 | Not detected | not detected |
| 271 | 46 | Spindle cell sarcoma | Myxoid | No | F | P | Soft Tissue | 2 | ROS1 | not detected |
| 272 | 81 | Myxofibrosarcoma | NA | No | F | LR | Soft Tissue | 2 | CTNNA2 | not detected |
| 273 | 54 | Chordoma | Conventional | No | M | LR | Bone | NA | MSH2, PIK3CA | not detected |
| 274 | 73 | Chondrosarcoma | Dedifferentiated central | No | F | P | Bone | NA | IDH1, TP53 | not detected |
| 275 | 73 | Osteosarcoma | Osteoblastic (arising on the background of a low-grade central osteosarcoma | No | M | P | Bone | 3 | RNF43, PIK3R1 | not detected |
| 276 | 85 | Myxofibrosarcoma | NA | No | F | P | Soft Tissue | 3 | Not detected | not detected |
| 277 | 11 | Osteosarcoma | Osteoblastic | No | F | P | Bone | 3 | FANCM | not detected |
| 278 | 44 | Liposarcoma | Pleomorphic | No | M | P | Soft Tissue | 3 | PIK3CA, TP53 | not detected |
| 279 | 45 | Leiomyosarcoma | Pleomorphic (metastases have heterologous osteoblastic/chondroblastic differentiation) | No | M | P | Soft Tissue | 3 | Not detected | not detected |
| 280 | 39 | Myxoinflammatory fibroblastic sarcoma | NA | No | F | P | Soft Tissue | 1 | Not detected | not detected |
| 281 | 21 | MPNST | Neurofibromatosis type 1 | No | M | P | Soft Tissue | 2 | Not detected | NF1^£^ |
| 282 | 32 | Epithelioid sarcoma | Classic | No | M | P | Soft Tissue | 3 | ATRX | not detected |
| 283 | 17 | Osteosarcoma | Osteoblastic | No | M | P | Bone | 3 | TP53, CTNNA2 | not detected |
| 284 | 70 | Myxofibrosarcoma | NA | No | F | Met | Soft Tissue | 3 | ATRX, TP53, | not detected |
| 285 | 9m | Spindle cell tumour | Low grade - difficult | No | F | P | Soft Tissue | 1 | Not detected | not detected |
| 286 | 16 | Osteosarcoma | Osteoblastic | No | F | P | Bone | 3 | Not detected | not detected |
| 287 | 19 | Osteosarcoma | Parosteal | No | F | P | Bone | 1 | PMS1 | not detected |
| 288 | 4 | Osteosarcoma | Osteoblastic | No | F | P | Bone | 3 | TSC1 | not detected |
| 289 | 24 | Osteosarcoma | Osteoblastic and chondroblastic | No | M | P | Bone | 3 | EGFR | not detected |
| 290 | 43 | Synovial sarcoma | Monophasic fibrous type | No | M | P | Soft Tissue | 2 | Not detected | not detected |
| 291 | 68 | Pleomorphic sarcoma | Undifferentiated | No | F | P | Soft Tissue | 3 | ATRX, TP53 | not detected |
| 292 | 50 | Atypical hybrid nerve sheath tumour (perineurioma/schwannoma) | NA | No | F | P | Soft Tissue | NA | Not detected | not detected |
| 293 | 65 | Phosphaturic mesenchymal tumour | NA | No | F | P | Bone | 1 | Not detected | not detected |
| 294 | 70 | Chondrosarcoma | Extraskeletal myxoid | No | M | Met | Soft Tissue | 3 | FANCI | not detected |
| 295 | 72 | Synovial sarcoma | Monophasic fibrous type | No | F | P | Soft Tissue | 3 | RB1, ARAF | not detected |
| 296 | 47 | Low grade fibromyxoid sarcoma | NA | No | F | P | Soft Tissue | 1 | Not detected | not detected |
| 297 | 49 | Chondrosarcoma | Dedifferentiated central | No | M | P | Bone | 3 | FANCD2, PIK3CA, PTEN, TP53, RICTOR, GNAS* | not detected |
| 298 | 25 | Chondrosarcoma | Conventional central | No | M | LR | Bone | 2 | IDH1, NF1 | not detected |
| 299 | 77 | Chordoma | Focal areas of dedifferentiated | No | M | Met | Bone | NA | Not detected | not detected |
| 300 | 41 | MPNST | Conventional | No | F | P | Soft Tissue | 3 | NF1, TP53 | NF1^£^ |
| 301 | 79 | Chondrosarcoma | Conventional central | No | F | P | Bone | 1 | IDH1 | not detected |
| 302 | 29 | Spindle cell sarcoma | High grade | No | M | P | Soft Tissue | 3 | Not detected | not detected |
| 303 | 67 | Myxofibrosarcoma | NA | No | F | P | Soft Tissue | 2 | ATM, ATRX, FGF6, MUTYH | not detected |
| 304 | 56 | Synovial sarcoma | Monophasic | No | M | P | Soft Tissue | 2 | Not detected | not detected |
| 305 | 84 | Myxofibrosarcoma | NA | No | M | P | Soft Tissue | 3 | ATRX, EGFR | not detected |
| 306 | 77 | Chondrosarcoma | Conventional central | No | M | P | Bone | 2 | IDH1, TP53 | not detected |
| 307 | 29 | Neurofibroma | NA | No | M | P | Soft Tissue | NA | Not detected | NF1^£^ |
| 308 | 19 | Osteosarcoma | Periosteal | No | M | P | Bone | 2 | Not detected | not detected |
| 309 | 20 | Osteosarcoma | Osteoblastic and fibroblastic | No | M | P | Bone | 3 | Not detected | not detected |
| 310 | 72 | Pleomorphic sarcoma | Undifferentiated | No | M | P | Soft Tissue | 3 | Not detected | not detected |
| 311 | 62 | Osteosarcoma | Soft Tissue | No | M | P | Soft Tissue | 3 | NF1 , FANCF | not detected |
| 312 | 74 | Myxofibrosarcoma | NA | No | F | P | Soft Tissue | 3 | Not detected | not detected |
| 313 | 38 | Ewing sarcoma | NA | No | M | P | Soft Tissue | 3 | Not detected | not detected |
| 314 | 39 | Solitary fibrous tumour | NA | No | M | P | Soft Tissue | NA | TP53 | not detected |
| 315 | 70 | Liposarcoma | Dedifferentiated | No | M | P | Soft Tissue | 3 | Not detected | not detected |
| 316 | 32 | Dermatofibrosarcoma protuberans | Fibrosarcomatous transformation | No | M | P | Soft Tissue | 3 | Not detected | not detected |
| 317 | 50 | Chondrosarcoma | Extraskeletal myxoid | No | F | P | Soft Tissue | 2 | Not detected | not detected |
| 318 | 12 | Ewing sarcoma | NA | No | M | P | Soft Tissue | 3 | SMARCA4 | not detected |
| 319 | 34 | Synovial sarcoma | Monophasic fibrous type | No | F | P | Soft Tissue | 2 | Not detected | not detected |
| 320 | 35 | MPNST | Conventional | EWSR1-NFACT2 rearranged sarcoma | M | P | Soft Tissue | 2 | Not detected | not detected |
| 321 | 69 | Liposarcoma | Pleomorphic | No | M | P | Soft Tissue | 3 | ATRX | TP53 |
| 322 | 22 | Synovial sarcoma | Biphasic | No | M | LR | Bone | 2 | Not detected | not detected |
| 323 | 35 | Osteosarcoma | Parosteal | No | M | P | Bone | 1 | RET | not detected |
| 324 | 15 | Osteosarcoma | Chondroblastic | No | M | P | Bone | 3 | Not detected | not detected |
| 325 | 72 | Liposarcoma | Dedifferentiated; Rhabdomyoblastic differentiation | No | F | P | Soft Tissue | 3 | Not detected | not detected |
| 326 | 66 | Myxofibrosarcoma | NA | No | M | P | Soft Tissue | 2 | Not detected | not detected |
| 327 | 29 | Spindle cell sarcoma | Pleomorphic | No | M | P | Soft Tissue | 2 | Not detected | not detected |
| 328 | 35 | Synovial sarcoma | Monophasic | No | F | P | Soft Tissue | 2 | Not detected | not detected |
| 329 | 66 | Spindle cell sarcoma | NA | No | F | P | Soft Tissue | 1 | RB1 | not detected |
| 330 | 66 | Chondrosarcoma | Conventional central | No | M | P | Bone | 2 | IDH1 | not detected |
| 331 | 84 | Myxofibrosarcoma | NA | No | F | P | Soft Tissue | 2 | ATRX, FRS2 | not detected |
| 332 | NA | Low grade fibromyxoid sarcoma | NA | No | F | LR | Soft Tissue | 1 | Not detected | not detected |
| 333 | 64 | Rhabdomyosarcoma | Pleomorphic | No | F | P | Soft Tissue | 3 | Not detected | not detected |
| 334 | 37 | Pleomorphic sarcoma | Pleomorphic | No | M | P | Bone | 3 | Not detected | not detected |
| 335 | 64 | Myxofibrosarcoma | NA | No | F | P | Soft Tissue | 3 | KRAS | not detected |
| 336 | 41 | Chondrosarcoma | Conventional central | No | M | P | Bone | 2 | IDH1 | not detected |
| 337 | 32 | Epithelioid haemangioendothelioma | NA | No | M | P | Soft Tissue | 2 | Not detected | not detected |
| 338 | 52 | Low grade fibromyxoid sarcoma | NA | No | F | P | Soft Tissue | 1 | Not detected | not detected |
| 339 | 85 | Chondrosarcoma | Conventional central | Arising in malignant synovial chondromatosis (ACVR2A-FN1) | F | P | Bone | 2 | Not detected | not detected |
| 340 | 11 | Ewing sarcoma | NA | No | M | P | Bone | 4 | NRAS, CDKN2A | not detected |
| 341 | 68 | Chondrosarcoma | Conventional central | No | M | P | Bone | 2 | IDH1 | not detected |
| 342 | 40 | Spindle cell sarcoma NOS | High grade | No | M | P | Bone | 3 | Not detected | not detected |
| 343 | 67 | MPNST | Conventional | No | F | P | Soft Tissue | 3 | NRAS, MDM2 | not detected |
| 344 | 26 | Ewing sarcoma | NA | No | M | P | Bone | 4 | Not detected | not detected |
| 345 | 42 | Liposarcoma | Pleomorphic | No | M | P | Soft Tissue | 3 | Not detected | not detected |
| 346 | 44 | Chondrosarcoma | Mesenchymal | Acral fibroblastic spindle cell neoplasm (EWSR1-SMAD3 rearranged) | M | P | Bone | 3 | Not detected | not detected |
| 347 | 53 | Myofibroblastic sarcoma | NA | No | M | P | Soft Tissue | 3 | Not detected | not detected |
| 348 | 40 | MPNST | Osteosarcomatous differentiation | No | M | P | Soft Tissue | 3 | Not detected | not detected |
| 349 | 55 | Myxofibrosarcoma | NA | No | F | P | Soft Tissue | 2 | TP53, RB1 | not detected |
| 350 | 66 | Liposarcoma | High grade | No | M | P | Soft Tissue | 3 | TP53, FGF9 | not detected |
